# Comparative outcomes of laparoscopic lateral suspension, sacrocolpopexy, and transvaginal mesh for advanced apical prolapse: A retrospective cohort study

**DOI:** 10.1371/journal.pone.0332526

**Published:** 2025-09-12

**Authors:** Lilu Guo, Xiaodi Li, Huihua Li, Binan Wang, Haichun Guo, Jingni Wu

**Affiliations:** 1 Department of Obstetrics and Gynecology, Changsha Maternal and Child Health Care Hospital, Changsha, China; 2 Department of Obstetrics and Gynecology, The Second Xiangya Hospital, Central South University, Changsha, China; IRCCS Burlo Garofolo Trieste, ITALY

## Abstract

**Objective:**

To compare perioperative outcomes and long-term anatomical/functional efficacy of laparoscopic lateral suspension (LLS), laparoscopic sacrocolpopexy (LSC), and transvaginal mesh (TVM) procedures in women with POP-Q stage III–IV apical prolapse.

**Methods:**

This retrospective cohort included 98 participants undergoing surgical repair between 1/1/2021 and 30/12/2021: 34 TVM, 35 LSC, and 29 LLS. Concomitant hysterectomy or uterine preservation was performed based on clinical indications. Anatomical outcomes were assessed via Pelvic Organ Prolapse Quantification (POP-Q) measurements, while functional outcomes and quality of life (QoL) were evaluated using Pelvic Floor Distress Inventory Questionnaire (PFDI-20) and Pelvic Floor Impact Questionnaire (PFIQ-7) questionnaires preoperatively and at 2-year follow-up. Multivariable regression adjusted for age, BMI, parity, and surgical approach.

**Results:**

LLS demonstrated superior perioperative outcomes, including shorter operative time (3.07 ± 0.15 vs. 4.59 ± 0.13 hours for LSC, p < 0.05), reduced blood loss (64.48 ± 4.62 vs. 116.18 ± 8.10 mL for TVM, p < 0.05), and shorter hospitalization (5.17 ± 0.20 vs. 6.21 ± 0.27 days for TVM, p < 0.05). Groin pain incidence was higher in TVM (21% vs. 0% in LSC/LLS, p < 0.05). All groups achieved significant anatomical restoration (POP-Q points p < 0.001) and QoL improvements (PFDI-20: TVM 97.31 → 8.37, LSC 108.92 → 5.76, LLS 110.89 → 6.64; PFIQ-7: TVM 103.86 → 3.45, LSC 113.24 → 9.28, LLS 122.99 → 8.04; p < 0.001). No intergroup differences persisted after adjusting confounders. Notably, TVM participants with uterine preservation reported significantly better PFIQ-7 scores than hysterectomy subgroups (0.96 ± 0.52 vs. 6.60 ± 3.46, p < 0.05), whereas LSC/LLS showed no such disparity.

**Conclusion:**

LLS, LSC, and TVM effectively restore anatomy and QoL in advanced apical prolapse, with LLS offering optimal perioperative safety. Uterine preservation during TVM enhances postoperative satisfaction, suggesting individualized surgical planning is critical. Long-term complications and durability require further investigation.

## Introduction

Pelvic organ prolapse (POP) is a prevalent condition affecting 30–50% of women, characterized by the descent of pelvic organs (e.g., bladder, uterus, rectum) due to weakened pelvic floor support [1]. POP is typically classified into anterior, posterior, and apical prolapse based on the organ involved. Apical prolapse, involving the vaginal vault or uterus, represents a complex subtype requiring surgical intervention in advanced stages (POP-Q III–IV) [2,3]. Laparoscopic sacrocolpopexy (LSC) and transvaginal mesh procedures (TVM), which are commonly employed surgical approach for the apical prolapse, as well as a new surgical approach lateral suspension (LLS), were creatively included in our study. Each of these methods has distinct advantages and challenges. While LSC is recognized for its durability and low recurrence rates [4], TVM—once widely adopted for its technical simplicity—has faced declining use due to mesh-related complications [5]. Emerging techniques like LLS offer shorter operative times and reduced morbidity but lack long-term quality of life (QoL) evaluation data [6].

A critical debate in POP repair revolves around concomitant hysterectomy versus uterine preservation. Proponents of uterine preservation argue that retaining the uterus minimizes surgical trauma, reduces operative time, and preserves anatomical integrity, potentially lowering mesh erosion risks [7]. Conversely, opponents emphasize the theoretical risk of undetected uterine pathology and long-term prolapse recurrence [8,9]. Existing studies, however, are limited by small cohorts, short follow-up, or a focus on anatomical outcomes without integrating participant-reported QoL measures [10–17]. Furthermore, no prior research has systematically compared uterine preservation outcomes across LLS, LSC, and TVM within a single cohort.

This study addresses these gaps by evaluating perioperative safety, anatomical restoration, and QoL improvements in 98 participants undergoing LLS, LSC, or TVM for advanced apical prolapse, with a 2-year follow-up. We further explore the impact of uterine preservation on functional outcomes—a novel aspect of this analysis. Our findings aim to guide evidence-based surgical decision-making and highlight the need for individualized treatment strategies in POP management.

## Materials and methods

The study included 98 participants with uterine prolapse stage III-IV who underwent POP surgery between 1/1/2021 and 30/12/2021. Medical records of participants who underwent surgical repair were reviewed between 1/12/2024 and 1/3/2025. Exclusion criteria comprised of surgical contraindications, previous prolapse repair, history of pelvic inflammatory disease, malignancy, psychiatric disorders, cognitive impairment, and incomplete medical records precluding adequate data abstraction. All surgical procedures were performed by experienced gynecologists. The study was granted by the Changsha Maternal and Child Health Care Hospital in accordance with the Declaration of Helsinki (EC-20241126-08). Written informed consent was obtained from each participant during their initial treatment, which included permission to use their anonymized medical records for future research. The individuals pictured in Figs S1 to S6 in S1 File have provided written informed consents (as outlined in PLOS consent form) to publish their images alongside the manuscript. All personal identifiers were deleted from the dataset to ensure confidentiality.

The stage of pelvic floor prolapse was assessed using a Pelvic Organ Prolapse Quantification (POP-Q) system. The gynecologist recorded the single most distal prolapse point of three (anterior, posterior or apical) compartments of the vagina from the hymen [18]. The participant’s QoL during the pre- and post-operative periods was assessed using the Pelvic Floor Distress Inventory Questionnaire (PFDI-20) and Pelvic Floor Impact Questionnaire (PFIQ-7) [19]. The PFDI-20 and PFIQ-7 questionnaires each consist of three subscales [20]. PFDI-20 reflects the different perspectives of prolapse-related symptoms (Pelvic Organ Prolapse Distress Inventory-6, POPDI-6), bowel problems (Colorectal-Anal Distress Inventory-8, CRADI-8), and urinary symptoms (UDI-6: Urinary Distress Inventory-6, UDI-6). PFIQ-7 describes the different impacts on participants’ quality of life related to urinary tract (Urinary Impact Questionnaire-7, UIQ-7), bowel or rectal (Colorectal-Anal Impact Qestionnaire-7, CRAIQ-7), and vaginal or pelvic symptoms (Pelvic Organ Prolapse Impact Questionnaire-7, POPIQ-7) [21]. For both questionnaires, items are scored on a 4-point scale (1 = not at all, 2 = somewhat, 3 = moderately, 4 = quite a bit in PFDI-20 and 0 = not at all, 1 = somewhat, 2 = moderately, 3 = quite a bit in PFIQ-7). Each subscale score was converted to a 0–100 scale by multiplying the mean item response by 25 (PFDI-20) or 100/3 (PFIQ-7). 3 subscale scores were added together to obtain the total PFDI-20 score or the total PFIQ-7 score (both range from 0 to 300). Higher scores on both questionnaires indicate greater symptom distress and greater negative impact on quality of life [22–24]. The surgical treatment and participant preoperative characteristics (participant’s age, BMI, parity status, pre-operative POP scores) were derived from the institution’s electronic medical record. Intraoperatively, participants were assessed for operative time, blood loss, and complications. Postoperative appointment follow-up was arranged at 2 years for evaluation of the impact of surgery on participant’s clinical symptoms and QoL, and the data of postoperative POP scores, PFDI-20 score, and PFIQ-7 score were collected.

The operations were divided into three groups: TVM, LSC and LLS. All procedures were performed according to standardized techniques. TVM: A customized polypropylene mesh was trimmed into a dragonfly-shaped configuration for anterior compartment reconstruction, supplemented by two mesh strips for sacrospinous ligament fixation: superior arms externalized through obturator membrane at pubic ramus-urethral meatus junction, inferior arms 1 cm lateral/caudal emerging anterior to ischial spine; sacrospinous fixation strips were anchored 1 cm medial to ischial spine and externalized 3 cm lateral/inferior to anal margin (S1 and S2 Figs in S1 File). LSC: Y-shaped mesh was laparoscopically secured to anterior/posterior vaginal fascia or the cervical stroma, with the long arm fixated tension-free to anterior longitudinal ligament of the sacral promontory (S3 and S4 Figs in S1 File). LLS: A tongue-configured mesh was suspended bilaterally through 45°peritoneal tunnels lateral to round ligaments, with arms externalized at points 4 cm superior and 3 cm lateral to anterior superior iliac spine (S5 and S6 Figs in S1 File). All meshes were secured with non-absorbable sutures at fixation points, followed by peritoneal closure with absorbable sutures. Surgical procedure selection (TVM, LSC, or LLS) was based on a combination of factors including participant age, desire for uterine preservation and participant preference after detailed counseling regarding the risks, benefits, and expected outcomes of each approach. Older participants often opted for TVM due to its perceived lower invasiveness. Younger participants frequently preferred LSC/LLS for its vaginal axis preservation. In line with the primary focus on apical support restoration in this study, concomitant posterior compartment defects were addressed with site-specific fascial repair (colporrhaphy) rather than placement of a separate posterior mesh. This approach aimed to minimize dissection, operative time, and potential mesh-related complications in the posterior compartment, particularly considering the primary indication was advanced apical prolapse. The decision was based on surgeon assessment of the posterior defect severity and the principle of minimizing foreign material where native tissue repair was deemed sufficient. Details of the surgical procedures were provided in the Supplementary Appendix following the literature and the actual situation in our hospital [25–27].

Analysis was performed with IBM SPSS 24.0 (IBM Corp., Armonk, NY, USA). *p* < 0.05 was accepted as statistically significant. Continuous variables with normal distribution were presented with means ± standard error (SE), continuous variables with abnormal distribution were expressed as medians and interquartile ranges, and categorical variables were expressed as numbers and percentages. Two independent groups with continuous variables were analyzed using Mann-Whitney test, two paired samples were analyzed using Wilcoxon signed-rank test and three independent groups were analyzed using Kruskal-Wallis test. Groups with categorical variables were tested using a Fisher’s exact test. The inclusion of both transvaginal and laparoscopic approaches reflected real-world individualized treatment. To isolate the effect of surgical techniques from inherent pathway differences, we applied multivariable linear regression analyses to control for potential confounders, including not only age, BMI, parity status and pre-operative scores, but also surgical approach.

## Results

### Participant characteristics

A total of 98 participants with stage III–IV apical prolapse underwent surgical repair: 34 TVM, 35 LSC, and 29 LLS. Baseline demographics and clinical characteristics are summarized in Table 1. Participants in the TVM group were significantly older than those in LSC and LLS groups (median age: 64 vs. 53 vs. 58 years, *p* < 0.05). No significant differences were observed in BMI, parity, or preoperative POP-Q measurements among groups (*p* ≥ 0.05).

**Table 1:** Characteristics of the study population.

Variables	TVM	LSC	LLS	P value		
	n = 34	n = 35	n = 29	LLS vs. TVM	LLS vs. LSC	LSC vs. TVM
Age (years)	64 (61-67)	53 (48-57)	58 (57-61)	<0.05	<0.05	<0.05
BMI, kg/m^2^	24.25 ± 0.39	25.1 ± 0.51	23.39 ± 0.41	NS		
Gravidity	2 (2-3)	2 (1-2)	2 (1-2)	NS		
Parity	3 (2-5)	3 (2-4)	3 (2-3)	NS		
Abortion	1 (0-2)	0 (0-2)	1 (0-2)	NS		
Aa	1.22 ± 0.22	0.68 ± 0.21	1.21 ± 0.26	NS		
Ba	3.02 ± 0.18	2.59 ± 0.25	2.79 ± 0.26	NS		
C	1.90 ± 0.39	3.39 ± 0.20	2.47 ± 0.40	NS	NS	<0.05
Ap	−0.62 ± 0.23	−0.57 ± 0.19	−1.02 ± 0.20	NS		
Bp	−0.37 ± 0.28	−0.40 ± 0.25	−0.67 ± 0.26	NS		
TVL	6.88 ± 0.07	6.90 ± 0.07	6.78 ± 0.08	NS		
PFDI	93.88 ± 4.92	109.80 ± 7.19	110.89 ± 6.98	NS		
PFIQ	101.05 ± 6.70	114.62 ± 6.85	122.99 ± 7.95	NS		

Values are considered significant if P < 0.05. Age, Gravidity, Parity and Abortion is expressed as a median (interquartile range). Others were expressed as mean ± standard error. NS: Not significant, LLS: laparoscopic lateral suspension, LSC: laparoscopic sacrocolpopexy, TVM: transvaginal mesh, PFDI: Pelvic Floor Distress Inventory Questionnaire and PFIQ: Pelvic Floor Impact Questionnaire.

### Comparison of perioperative indicators of three surgical methods

LLS demonstrated supreme perioperative outcomes, including the shortest operative time (3.07 ± 0.15 hours vs. LSC: 4.59 ± 0.13 hours, *p* < 0.05), minimal blood loss (64.48 ± 4.62 mL vs. TVM: 116.18 ± 8.10 mL, *p* < 0.05), and reduced hospitalization duration (5.17 ± 0.20 days vs. TVM: 6.21 ± 0.27 days, *p* < 0.05). TVM was associated with prolonged catheterization (70.24 ± 4.58 hours vs. LSC/LLS: 53.23 ± 2.77 and 51.55 ± 2.59 hours, *p* < 0.05) and higher groin pain incidence (21% vs. 0% in LSC/LLS, *p* < 0.05). Complication rates (fever, urinary retention, mesh exposure) were comparable across groups (*p* ≥ 0.05) (Table 2).

**Table 2:** Comparison of perioperative indicators of the three pelvic organ prolapse surgeries.

Perioperative indicators	TVM	LSC	LLS	P value		
	n = 34	n = 35	n = 29	LLS vs. TVM	LLS vs. LSC	LSC vs. TVM
**Duration of surgery (h)**	2.81 ± 0.13	4.59 ± 0.13	3.07 ± 0.15	NS	<0.05	<0.05
**Amount of blood loss (mL)**	116.18 ± 8.10	87.14 ± 7.20	64.48 ± 4.62	<0.05	<0.05	<0.05
**Indwelling catheter time (h)**	70.24 ± 4.58	53.23 ± 2.77	51.55 ± 2.59	<0.05	NS	<0.05
**Recovery time for exhaustion (h)**	22.38 ± 1.24	27.23 ± 1.83	26.38 ± 2.17	NS	NS	<0.05
**Duration of admission (days)**	6.21 ± 0.27	5.67 ± 0.15	5.17 ± 0.20	<0.05	NS	NS
**Complications during admission**	10	1	2			
Fever of unknown origin	1(3%)	1(3%)	1 (3%)	NS	NS	NS
Urinary Retention	2(6%)	0	1(3%)	NS	NS	NS
Groin pain	7(21%)	0	0	<0.05	NS	<0.05
mesh exposure	4	3	3	NS	NS	NS

LLS: laparoscopic lateral suspension, LSC: laparoscopic sacrocolpopexy, TVM: transvaginal mesh, NS: Not significant.

### Quantification of pelvic organ prolapse after surgery

Postoperative POP-Q measurements confirmed significant anatomical restoration across all surgical groups (*p* < 0.001), with no cases of apical prolapse recurrence during the 2-year follow-up. The anterior vaginal wall (Aa point) improved from 1.28 ± 0.20 to −2.74 ± 0.05 in the TVM group, 0.74 ± 0.21 to −2.64 ± 0.08 in the LSC group, and 1.21 ± 0.26 to −2.64 ± 0.09 in the LLS group. Similarly, the apical support (C point) demonstrated marked correction: TVM (1.90 ± 0.37 to −5.78 ± 0.06), LSC (3.33 ± 0.21 to −5.67 ± 0.07), and LLS (2.47 ± 0.40 to −5.81 ± 0.06). Posterior compartment parameters (Ap and Bp) followed comparable improvement trends, achieving near-normal anatomical position (Table 3).

**Table 3:** Pre- and post-operative (2-year follow-up) pelvic organ prolapse quantification.

Variables	TVM			LSC			LLS		
	Pre-operative assessment	Post- operative assessment	P value	Pre-operative assessment	Post-operative assessment	P value	Pre-operative assessment	Post-operative assessment	P value
Aa	1.28 ± 0.20	−2.74 ± 0.05	<0.001	0.74 ± 0.21	−2.64 ± 0.08	<0.001	1.21 ± 0.26	−2.64 ± 0.09	<0.001
Ba	3.12 ± 0.18	−2.84 ± 0.08	<0.001	2.62 ± 0.25	−2.71 ± 0.08	<0.001	2.79 ± 0.26	−2.72 ± 0.08	<0.001
C	1.90 ± 0.37	−5.78 ± 0.06	<0.001	3.33 ± 0.21	−5.67 ± 0.07	<0.001	2.47 ± 0.40	−5.81 ± 0.06	<0.001
Ap	−0.46 ± 0.25	−2.93 ± 0.04	<0.001	−0.59 ± 0.19	−2.93 ± 0.04	<0.001	−1.02 ± 0.20	−2.98 ± 0.02	<0.001
Bp	0.03 ± 0.34	−2.57 ± 0.23	<0.001	−0.41 ± 0.25	−2.89 ± 0.05	<0.001	−0.67 ± 0.26	−2.97 ± 0.03	<0.001

LLS: laparoscopic lateral suspension, LSC: laparoscopic sacrocolpopexy, TVM: transvaginal mesh.

### Comparison of quality of life after surgery

Functional outcomes, evaluated by comparing the pre- and post-operative scores, were illustrated in Table 4. PFDI-20 and PFIQ-7 scores showed significant improvements in the QoL at 2-year postoperative follow-up compared to the baseline score. The median value of PFDI-20 score decreased significantly from 97.31 ± 6.06 to 8.37 ± 3.29 after the TVM surgery, from 108.92 ± 7.03 to 5.76 ± 1.53 after the LSC surgery, and from 110.89 ± 6.98 to 6.64 ± 1.69 after the LLS surgery (*p* < 0.001). The median value of PFIQ-7 score decreased significantly from 103.86 ± 6.70 to 3.45 ± 1.60 after the TVM surgery, from 113.24 ± 6.79 to 9.28 ± 2.47 after the LSC surgery, and from 122.99 ± 7.95 to 8.04 ± 2.69 after the LLS surgery (*p* < 0.001).

**Table 4:** Pre- and post-operative (2-year follow-up) quality of life evaluation.

Variables	TVM			LSC			LLS		
	Pre-operative assessment	Post-operative assessment	P value	Pre-operative assessment	Post-operative assessment	P value	Pre-operative assessment	Post-operative assessment	P value
PFDI	97.31 ± 6.06	8.37 ± 3.29	<0.001	108.92 ± 7.03	5.76 ± 1.53	<0.001	110.89 ± 6.98	6.64 ± 1.69	<0.001
PFIQ	103.86 ± 6.70	3.45 ± 1.60	<0.001	113.24 ± 6.79	9.28 ± 2.47	<0.001	122.99 ± 7.95	8.04 ± 2.69	<0.001

LLS: laparoscopic lateral suspension, LSC: laparoscopic sacrocolpopexy, TVM: transvaginal mesh, PFDI: Pelvic Floor Distress Inventory Questionnaire and PFIQ: Pelvic Floor Impact Questionnaire.

### Comparison anatomical and functional outcomes of three surgical methods

As shown in S1 Table in S1 File, univariate analysis revealed that similar anatomical and functional outcomes were observed among TVM, LSC and LLS groups for the surgical treatment of uterine prolapse. When adjusted for age, BMI, gravidity, parity and abortion, postoperative POP-Q scores, PFDI-20 and PFIQ-7 scores showed no differences among the three groups. Even after adjusting for all the potential confounder factors, such as pre-operative POP-Q scores, transvaginal or laparoscopic surgical approach and hysterectomy rate, similar postoperative scores were observed among three groups.

### Comparison of surgeries with and without concomitant hysterectomy

Table 5 shows anatomical and functional outcomes 2 years after surgery with and without concomitant hysterectomy. Uterine preservation during TVM correlated with superior PFIQ-7 scores (0.96 ± 0.52 vs. 6.60 ± 3.46 with hysterectomy, *p* < 0.05), despite comparable anatomical outcomes. In contrast, LSC and LLS showed no functional or anatomical differences between hysterectomy and uterine preservation subgroups (*p* ≥ 0.05).

**Table 5:** Comparison of hysterectomy and uterine preservation in the three pelvic organ prolapse surgeries.

Variables	TVM			LSC			LLS		
	Hysterectomy (n = 15)	Uterine preservation (n = 19)	P Value	Hysterectomy (n = 27)	Uterine preservation (n = 8)	P value	Hysterectomy (n = 21)	Uterine preservation (n = 8)	P value
Aa	−2.67 ± 0.09	−2.79 ± 0.06	NS	−2.65 ± 0.08	−2.63 ± 0.18	NS	−2.62 ± 0.11	−2.69 ± 0.13	NS
Ba	−2.83 ± 0.18	−2.84 ± 0.05	NS	−2.70 ± 0.09	−2.75 ± 0.19	NS	−2.69 ± 0.11	−2.81 ± 0.09	NS
C	−5.80 ± 0.10	−5.76 ± 0.08	NS	−5.76 ± 0.07	−5.38 ± 0.13	NS	−5.81 ± 0.06	−5.81 ± 0.13	NS
Ap	−2.97 ± 0.03	−2.89 ± 0.06	NS	−2.93 ± 0.05	−2.94 ± 0.06	NS	−2.98 ± 0.02	−2.97 ± 0.01	NS
Bp	−2.90 ± 0.07	−2.32 ± 0.40	NS	−2.93 ± 0.05	−2.75 ± 0.16	NS	−2.95 ± 0.05	−2.96 ± 0.09	NS
PFDI	12.67 ± 7.01	4.98 ± 1.99	NS	5.46 ± 1.72	6.78 ± 3.52	NS	6.40 ± 1.92	7.29 ± 3.69	NS
PFIQ	6.60 ± 3.46	0.96 ± 0.52	<0.05	8.33 ± 2.70	12.50 ± 6.10	NS	7.93 ± 3.17	8.33 ± 5.45	NS

LLS: laparoscopic lateral suspension, LSC: laparoscopic sacrocolpopexy, TVM: transvaginal mesh, NS: Not significant, PFDI: Pelvic Floor Distress Inventory Questionnaire and PFIQ: Pelvic Floor Impact Questionnaire.

## Discussion

Our research uniquely compares the three surgical methods—TVM, LSC, and LLS—highlighting significant improvements in pelvic organ prolapse and QoL postoperatively. Notably, LLS exhibited a shorter operative time, reduced admission duration and better perioperative outcomes. What’s more, in the TVM group, participants retained their uterus reported higher QoL than that without uterus.

Regarding demographic differences, the age variation among groups stemmed from non-randomized selection, influencing surgical preferences. Older participants preferred TVM for its perceived lower invasiveness, while younger participants leaned towards LSC for its durability and minimal effects on sexual function [28,29]. Although perioperative outcomes were generally favorable across techniques, our study noted slightly higher groin pain in the TVM group, shorter operation and admission time in the LLS group without compromising results, and LLS and LSC groups had the advantage over TVM group of less blood loss during the operation and shorter indwelling catheter time, which were consistent with previous analyses [30–32]. As a new surgical treatment for apical prolapse, LLS has a greater advantage in our study. This is possibly because the operation field during laparoscopic surgery has a good exposure and the suspension area of LLS avoids the vascular and nerve danger, which simplify the operation and shortened the learning curve [31,32]. More participants felt groin pain in the TVM group which might be associated with stimulation after puncture, fascia traction by the mesh or pain caused by nerve compression. Unlike some researches, discrepancy could be attributed to various factors, including surgical technique, participant age, sexual activity level, mesh type, and repair area. After two years of follow-up, our analysis revealed significant improvements in both anatomical structure and QoL across all three surgical options, affirming their viability for treating apical prolapse. Even after adjusting for age and other confounding factors, we found no substantial differences in clinical outcomes or participants’ self-reported symptoms. Notably, advanced pelvic organ prolapse frequently presents with concurrent multi-compartment and multi-level defects. The three apical suspension techniques demonstrated equivalent efficacy in resolving concomitant anterior and posterior vaginal wall prolapse. These findings align with previous studies reporting comparable anatomical success rates across these procedures. For instance, a systematic review by Zhang et al. indicated that both LSC and TVM had similar anatomical success rates [33] and Lsenlik et al. suggested that LLS can serve as a safe, effective, and feasible alternative to LSC [34], which emphasizing their efficacy in addressing pelvic organ prolapse. However, some literature highlights differences in specific outcomes. For example, a meta-analysis by Maher et al. reported that LSC had a superior anatomical outcomes compared to TVM with lower reoperation rate, attributing this to the more robust support provided by the abdominal approach [35]. Conversely, our findings suggest that while LSC may offer distinct benefits, the overall clinical efficacy of the three methods remains largely equivalent in our cohort. Moreover, regarding QoL, our results are consistent with Maher’s conclusion that both TVM and LSC significantly improve QoL metrics postoperatively. However, this review also noted a higher incidence of complications such as mesh exposure with TVM, which may impact long-term QoL. In our study, while we observed no significant differences in complications among the techniques, the potential for higher mesh exposure in TVM warrants further investigation.

In our study, we found that participants in the TVM group who retained their uterus reported a significantly higher QoL compared to those who underwent hysterectomy. In contrast, for the LSC and LLS procedures, there were no significant differences in the participants’ QoL. What’s more, no differences of the anatomical outcomes were observed in the three groups. The higher PFIQ scores in TVM participants with uterine preservation may relate to reduced mesh-related complications (e.g., nerve compression) when avoiding hysterectomy, whereas LSC/LLS inherently involve less vaginal dissection, mitigating this effect. This finding is particularly noteworthy, as it suggests that the decision to preserve the uterus can have a meaningful impact on functional outcomes for women undergoing TVM surgery for apical prolapse. Previous literature has offered mixed insights regarding the impact of hysterectomy on surgical outcomes for pelvic organ prolapse. Consistent to our study, several studies indicate that uterine preservation may decrease the risk of mesh exposure and shorten the operation time without worsening the prolapse outcomes. In the TVM group, uterine preservation or hysterectomy do not affect the improvement of postoperative POP-Q values [7,36,37]. We further refined these studies and analyzed the functional outcomes in different surgical approaches. However, the sample size is limited and future multi-center studies with larger cohorts are needed to confirm these findings across diverse populations.

This study has some limitations. Our research was retrospective and has a relatively small number of cases. The non-randomized allocation to surgical groups introduces potential selection bias, although we adjusted for key confounders statistically. While our study provides valuable data with a median follow-up of 24 months, this time-frame may be insufficient to fully evaluate the long-term anatomical durability of the procedures, particularly regarding prolapse recurrence. Some recurrences may manifest beyond this period. Future studies with larger cohorts and extended follow-up durations (e.g., 5 years or longer) are necessary to comprehensively assess the longevity of the repair achieved with LLS, LSC, and TVM.

The innovation of our study is to directly compare the outcomes of the three surgical techniques while examining the effects of uterine preservation or not. By focusing on both anatomical and functional outcomes, we contribute to a more comprehensive understanding of how surgical choices can impact women’s lives after prolapse surgery. The finding that TVM participants retaining their uterus have a higher QoL underscores the importance of individualized surgical planning and participant-centered decision-making. Further studies are necessary to explore the long-term implications of these surgical choices, ultimately aiming to enhance participant satisfaction and clinical outcomes in pelvic organ prolapse management.

## Supporting information

S1 File **S1 Fig.** The mesh was placed between the bladder and the vaginal fascia in TVM. **S2 Fig.** The mesh was placed between the posterior vaginal wall and the rectal fascia in TVM. **S3 Fig.** The mesh was sutured to the anterior sacral ligament with nonabsorbable sutures in LSC. **S4 Fig.** The mesh was fixed to the anterior sacral ligament and embedded in the subperitoneum in LSC. **S5 Fig.** The mesh was placed between the bladder and the vaginal fascia in LLS. **S6 Fig.** The mesh was subperitoneal through the lateral extraperitoneal wall in LLS. **S1 Table. Multivariable linear regression analysis showing the efficacy of three surgical methods for pelvic floor prolapse.** Model 1 represents the univariate analysis of POP-Q values after TVM, LSC or LLS surgery. Multivariable linear regression analysis Model 2 included Model 1 plus the potential confounders of age, BMI, Gravidity, Parity and abortion. Model 3 included Model 2 plus the potential confounders of pre-operative scores (POP-Q, PFDI and PFIQ scores), transvaginal or laparoscopic surgical approach and hysterectomy rate. Values were considered significant if P < 0.05.
(DOCX)

S2 File Renamed_90fba. (ZIP)
