# Supplementary material for: Comparative outcomes of laparoscopic lateral suspension, sacrocolpopexy, and transvaginal mesh for advanced apical prolapse: A retrospective cohort study
Source: PLoS One. 2025 Sep 12;20(9):e0332526. doi: 10.1371/journal.pone.0332526 (PMC12431353; doi:10.1371/journal.pone.0332526)
Supplement: S1 File — S1 Fig. The mesh was placed between the bladder and the vaginal fascia in TVM. S2 Fig. The mesh was placed between the posterior vaginal wall and the rectal fascia in TVM. S3 Fig. The mesh was sutured to the anterior sacral ligament with nonabsorbable sutures in LSC. S4 Fig. The mesh was fixed to the anterior sacral ligament and embedded in the subperitoneum in LSC. S5 Fig. The mesh was placed between the bladder and the vaginal fascia in LLS. S6 Fig. The mesh was subperitoneal through the lateral extraperitoneal wall in LLS. S1 Table. Multivariable linear regression analysis showing the efficacy of three surgical methods for pelvic floor prolapse. Model 1 represents the univariate analysis of POP-Q values after TVM, LSC or LLS surgery. Multivariable linear regression analysis Model 2 included Model 1 plus the potential confounders of age, BMI, Gravidity, Parity and abortion. Model 3 included Model 2 plus the potential confounders of pre-operative scores (POP-Q, PFDI and PFIQ scores), transvaginal or laparoscopic surgical approach and hysterectomy rate. Values were considered significant if P < 0.05. (DOCX) [file pone.0332526.s001.docx]

Transvaginal Mesh Procedure (TVM)

The transvaginal mesh (TVM) procedure utilized a customized polypropylene mesh trimmed into a dragonfly-shaped configuration (7cm anterior-posterior×15cm lateral) for anterior compartment reconstruction, supplemented by two 1×15 cm mesh strips for sacrospinous ligament fixation. For anterior mesh placement: (1) Superior arms were externalized through trocar entry points at the urethral meatus level/lateral descending pubic ramus junction, traversing the obturator membrane 1 cm posterior to the pubic bone into the vesicovaginal space; (2) Inferior arms were externalized 1 cm lateral and 2 cm caudal to superior points, penetrating the obturator membrane inferior to the pubic ramus and emerging 1 cm anterior to the ischial spine. The mesh was tension-free positioned in the vesicovaginal space and secured to suburethral fascia/vaginal apex with 2-0 Ethibond sutures (S1 Figure). For posterior reconstruction: The sacrospinous ligament was accessed through a posterior vaginal incision, with mesh strips anchored to the vaginal apex/cervical fascia and traversing the ligament 1 cm medial to the ischial spine before externalization at skin points 3 cm lateral and 3 cm inferior to the anal margin (S2 Figure).

Laparoscopic Sacrocolpopexy (LSC)

The vesicovaginal space was dissected to the level of the urethrovaginal sulcus, followed by dissection of the rectovaginal space, the right pararectal space, and the presacral space. The short arms of the Y-shaped polypropylene mesh were sutured to the anterior and posterior vaginal wall fascia or the cervix stroma. The long arm of the Y-mesh was pulled upwards through the presacral space to the avascular area of the first sacral vertebra. A secure fixation point was identified, and the mesh was sutured tension-free to the anterior sacral ligament (S3 Figure). The mesh length was trimmed as needed. The mesh was closed with absorbable sutures and embedded in the retroperitoneum (S4 Figure).

Laparoscopic Lateral Suspension (LLS)

The left abdominal suspension point was located 4 cm above and 3 cm lateral to the left anterior superior iliac spine, with a corresponding point designated on the right side. Under laparoscopic guidance, the vesicovaginal space was dissected to the level of the urethrovaginal sulcus. The anterior pelvic floor repair system mesh (polypropylene material) was trimmed, removing the two lower branches and shaping the middle portion into a tongue, sized to correspond to the prolapsed section of the anterior vaginal wall. The top of the mesh's middle portion was sutured to the vaginal stump or anterior cervical ligament using non-absorbable sutures, and the tongue-shaped portion was fixed to the vaginal wall with absorbable sutures (S5 Figure). Under laparoscopic guidance, a curved needle was passed through the peritoneal layer (without penetrating it) and blunt dissection was performed to create a peritoneal tunnel at a 45° angle with the round ligament. The needle was pulled out beneath the bladder peritoneal fold (S6 Figure). The left arm of the mesh was clamped and pulled through the left abdominal suspension puncture site. The same procedure was applied on the right side to pull the right arm of the mesh. The mesh arms were adjusted according to the degree of prolapse to achieve the desired height. The peritoneum was closed with absorbable sutures, and any excess mesh was trimmed externally.
