# Supplementary material for: Comparative outcomes of laparoscopic lateral suspension, sacrocolpopexy, and transvaginal mesh for advanced apical prolapse: A retrospective cohort study
Source: PLoS One. 2025 Sep 12;20(9):e0332526. doi: 10.1371/journal.pone.0332526 (PMC12431353; doi:10.1371/journal.pone.0332526)
Supplement: S2 File — (ZIP) [file pone.0332526.s002.zip › S1 Table.docx]

**S1 Table. Multivariable linear regression analysis showing the efficacy of three surgical methods for pelvic floor prolapse**

| **Variables** | | Model 1 | Model 2 | Model 2 |
| --- | --- | --- | --- | --- |
|  |  | **P value** | **P value** | **P value** |
| Aa |  |  |  |  |
|  | LLS vs.LSC | NS | NS | NS |
|  | LLS vs.TVM | NS | NS | NS |
|  | LSC vs.TVM | NS | NS | NS |
| Ba |  |  |  |  |
|  | LLS vs.LSC | NS | NS | NS |
|  | LLS vs.TVM | NS | NS | NS |
|  | LSC vs.TVM | NS | NS | NS |
| C |  |  |  |  |
|  | LLS vs.LSC | NS | NS | NS |
|  | LLS vs.TVM | NS | NS | NS |
|  | LSC vs.TVM | NS | NS | NS |
| Ap |  |  |  |  |
|  | LLS vs.LSC | NS | NS | NS |
|  | LLS vs.TVM | NS | NS | NS |
|  | LSC vs.TVM | NS | NS | NS |
| Bp |  |  |  |  |
|  | LLS vs.LSC | NS | NS | NS |
|  | LLS vs.TVM | NS | NS | NS |
|  | LSC vs.TVM | NS | NS | NS |
| PFDI |  |  |  |  |
|  | LLS vs.LSC | NS | NS | NS |
|  | LLS vs.TVM | NS | NS | NS |
|  | LSC vs.TVM | NS | NS | NS |
| PFIQ |  |  |  |  |
|  | LLS vs.LSC | NS | NS | NS |
|  | LLS vs.TVM | NS | NS | NS |
|  | LSC vs.TVM | NS | NS | NS |

Model 1 represents the univariate analysis of POP-Q values after TVM, LSC or LLS surgery. The multivariable linear regression Model 2 included Model 1 plus the potential confounders of age, BMI, Gravidity, Parity and abortion. Model 3 included Model 2 plus the potential confounders of pre-operative scores (POP-Q, PFDI and PFIQ scores), transvaginal or laparoscopic surgical approach and hysterectomy rate. Values were considered significant if P<0.05.
