# Supplementary figures and images for: Comparative outcomes of laparoscopic lateral suspension, sacrocolpopexy, and transvaginal mesh for advanced apical prolapse: A retrospective cohort study
Source: PLoS One. 2025 Sep 12;20(9):e0332526. doi: 10.1371/journal.pone.0332526 (PMC12431353; doi:10.1371/journal.pone.0332526)

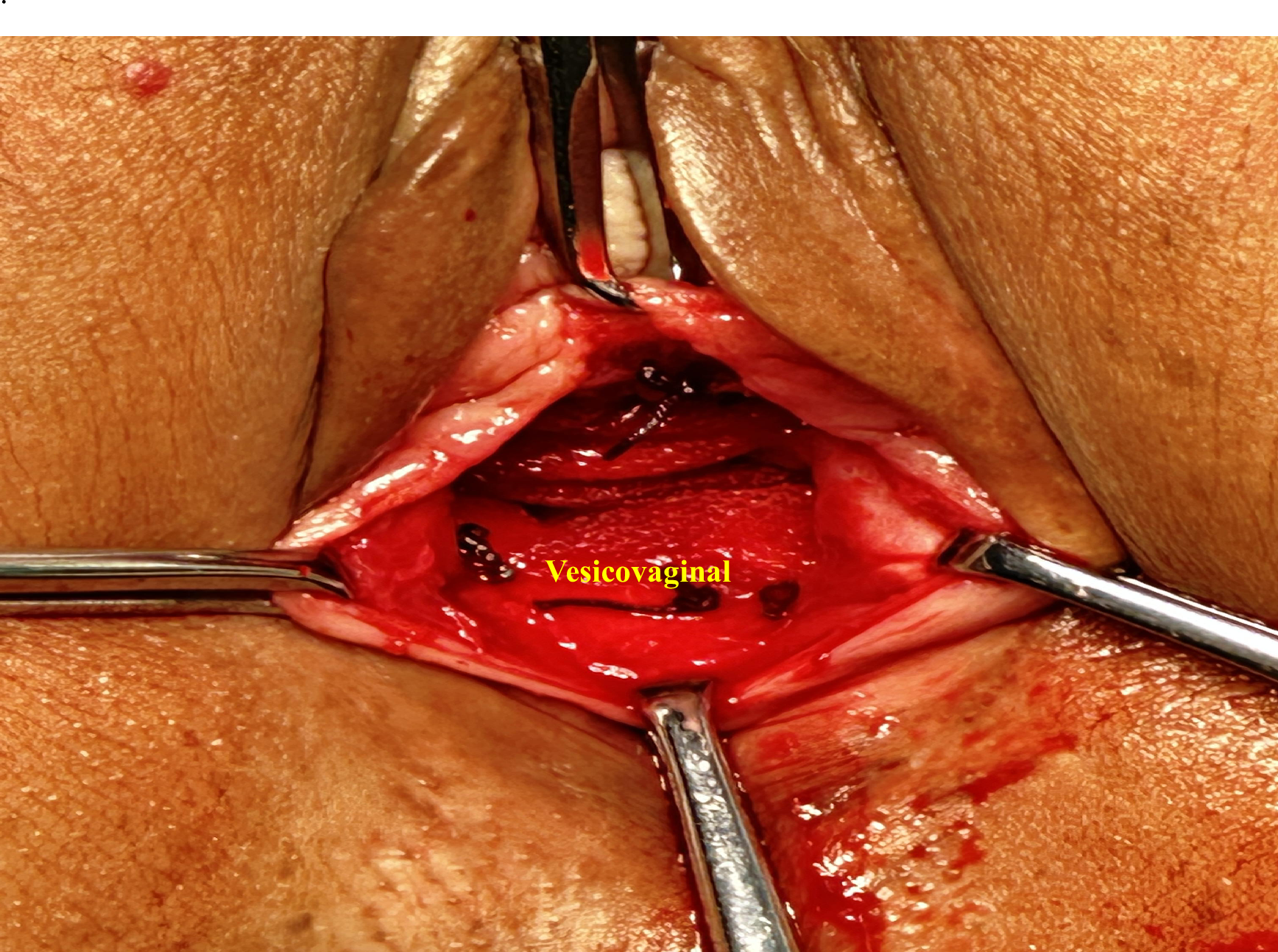

Supplement: S2 File — (ZIP) [file pone.0332526.s002.zip › S1_Fig.tif]

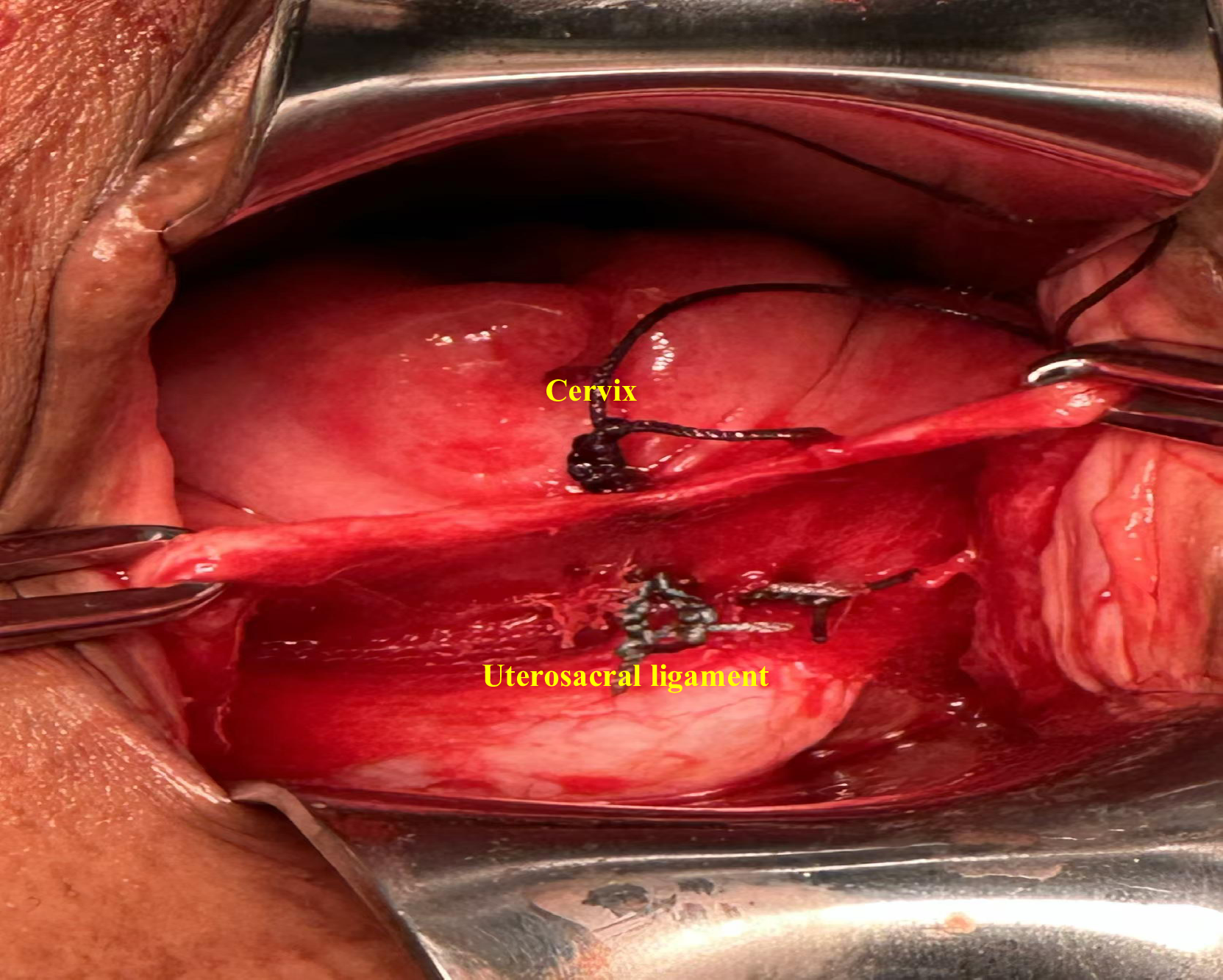

Supplement: S2 File — (ZIP) [file pone.0332526.s002.zip › S2_Fig.tif]

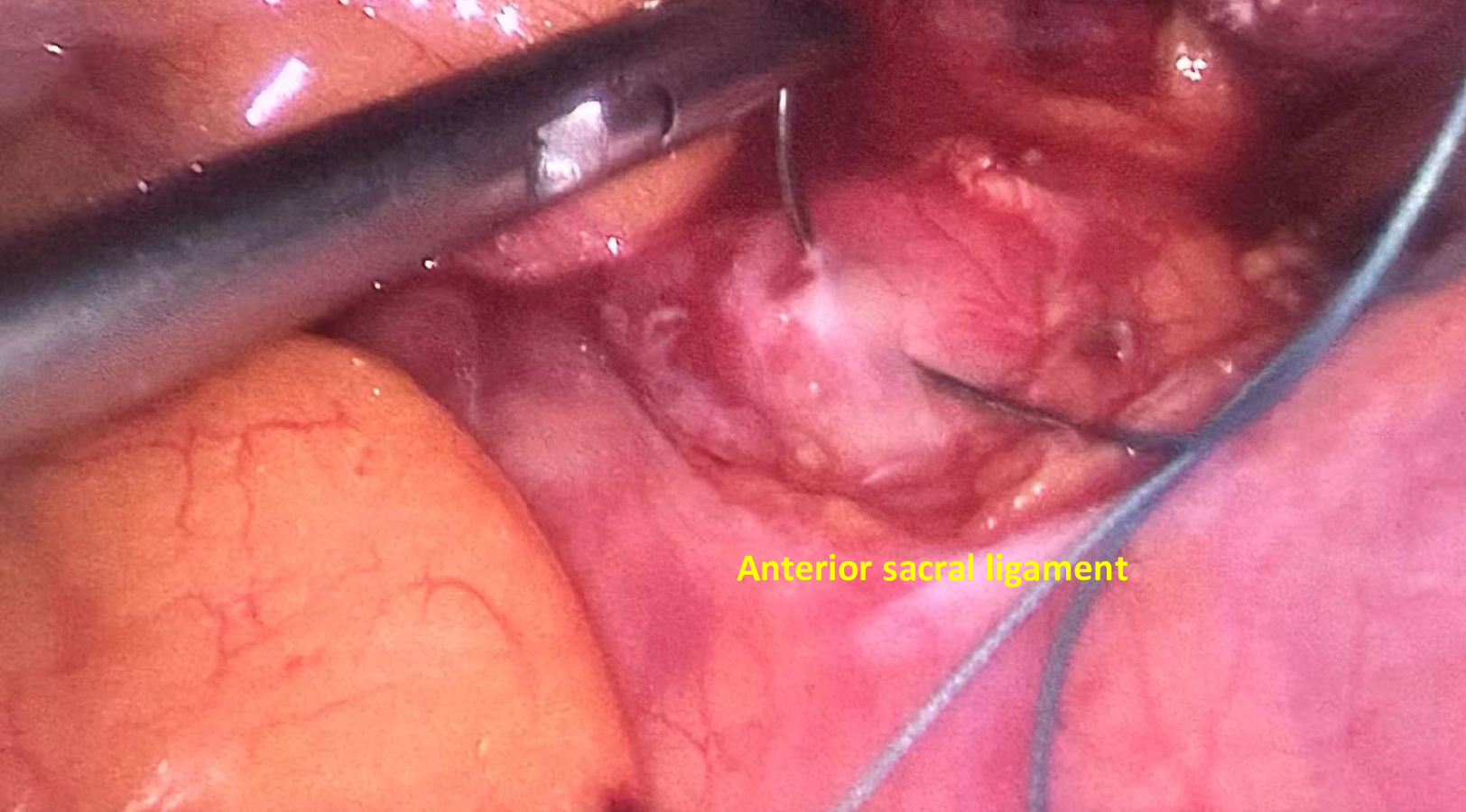

Supplement: S2 File — (ZIP) [file pone.0332526.s002.zip › S3_Fig.tif]

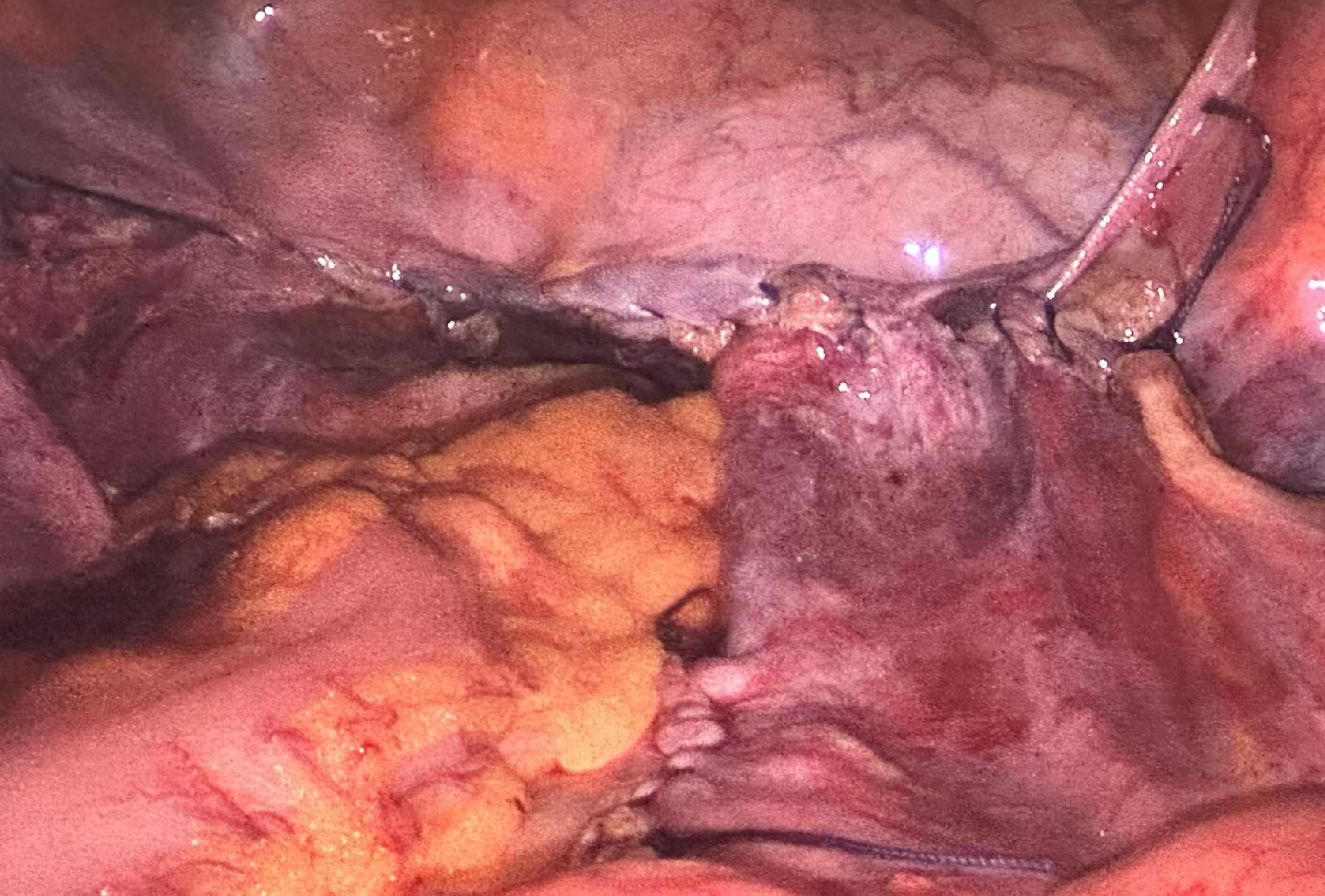

Supplement: S2 File — (ZIP) [file pone.0332526.s002.zip › S4_Fig.tif]

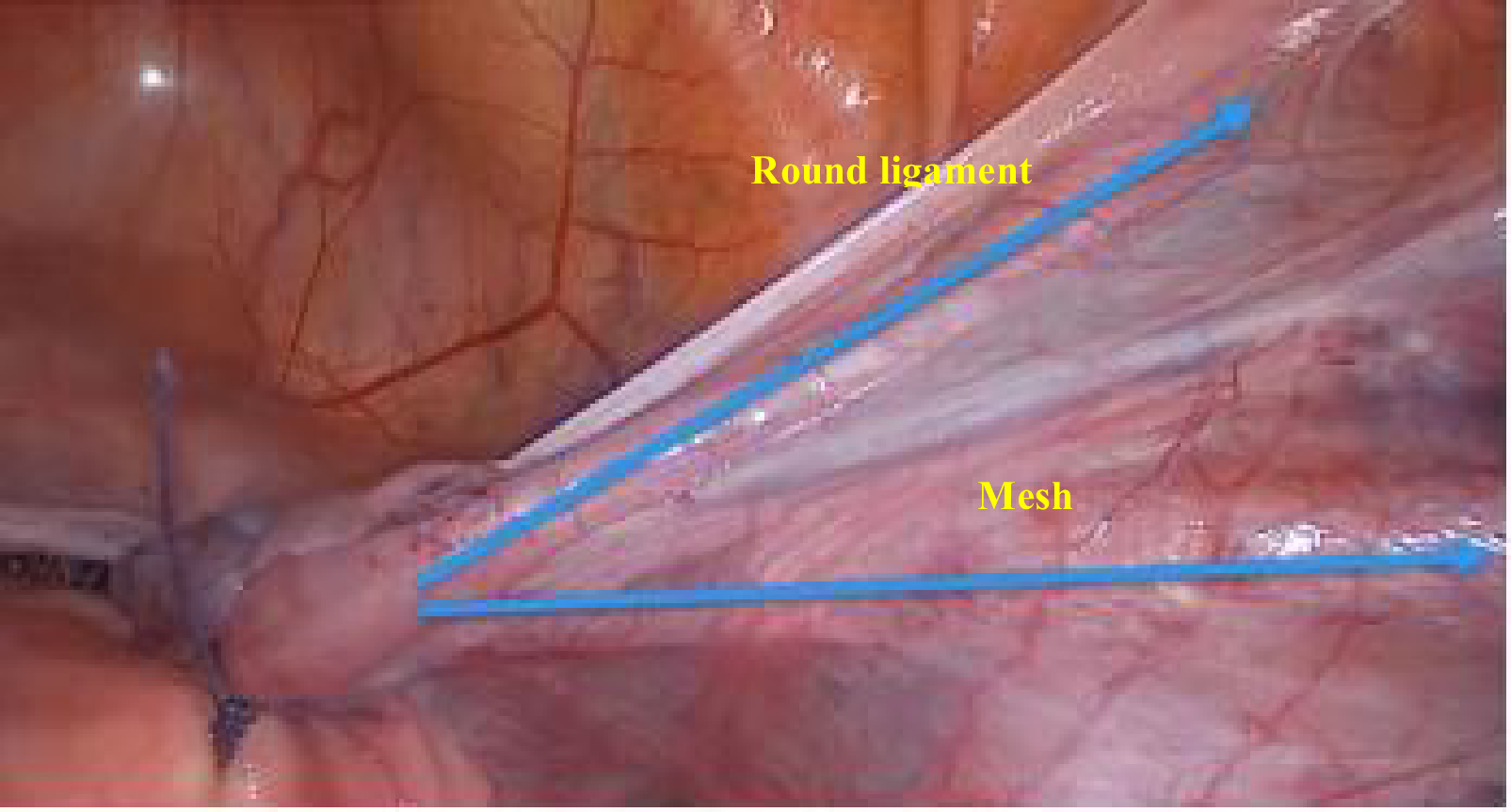

Supplement: S2 File — (ZIP) [file pone.0332526.s002.zip › S6_Fig.tif]

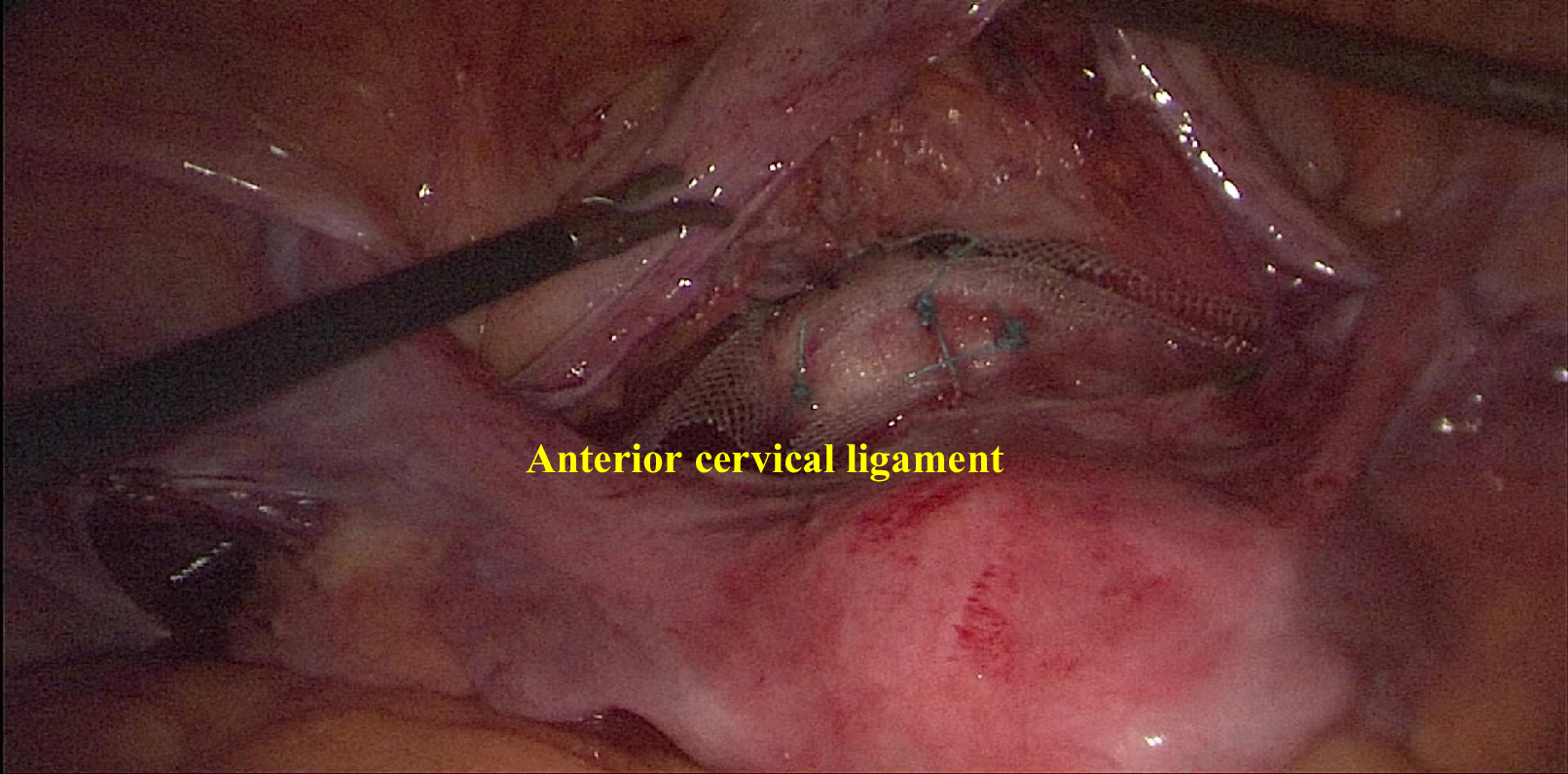

Supplement: S2 File — (ZIP) [file pone.0332526.s002.zip › S5_Fig.tif]
